# The Therapeutic Efficiency of Oral Preparations

**Published:** 1916-08

**Authors:** Hermann Prinz

**Affiliations:** Philadelphia, Pa.


### THE THERAPEUTIC EFFICIENCY OF ORAL PREPARATIONS.

BY HERMANN PRINZ, A.M., M.D., D.D.S., PHILADELPHIA, PA.

Oral hygiene involves the preservation of the normal equilibrium of the oral cavity and its contents. The remedies intended for the maintenance of the health of the soft structures of the mouth and the teeth may be conveniently divided into those prescribed for specific diseased conditions and those employed as hygienic measures for daily use. In the mouths of most civilized races, the mucous membrane, on account of the present perverted methods of preparing and seasoning our food-stuffs, is found more or less always in a state of mild chronic inflammation, while the hard structures of the oral cavity, the teeth, are subjected to a process of molecular destruction known as dental caries. Dental caries is not a disease in the same strict sense of the word in which the latter term is usually applied, but is "a process distinctly allied, both in its chemic and bacteriologic aspects, to the general phenomena of putrefaction" (Goadby). While certain preliminary intrinsic causes, *i. e.*, anomalies of position, outline and structure, etc., may profoundly alter the predisposition of the tooth to carious destruction as a whole or in part, dental caries will always occur if a tooth is subjected to the influences of suitable environments, and it does not matter whether the tooth forms an integral part of the anatomy of the individual, or whether it is separated wholly or in part from its original owner. Miller formulated an explanation of the nature of the carious process which defines this phenomenon as: a chemico-parasitic process consisting of two definite states, *i. e.*, the decalcification of the tissues and the dissolution of the remaining organic matrix. In caries of the enamel, the latter phenomenon is not observed on account of the minute quantities of organic matter contained therein. The accumulation of carbohydrate food debris on and about the teeth is held directly responsible as being the incipient factor in the production of the decalcifying agents. The direct or indirect splitting up of these carbohydrates by fission fungi into acids, *i. e.*, principally lactic acid, furnishes the attacking agent which decalcifies the enamel.

The hygienic care of the mouth intends primarily to keep the mucous membrane and the teeth in a state of healthy equilibrium by overcoming the above enumerated morbific processes. Nature has instituted protective measures of her own to accomplish the desired end. The normal mouth is fairly well protected against the continual onslaughts of the omnipresent bacteria through an unusually rich blood supply of the oral tissues, a high resistance of their epithelial lining, and a free flow of saliva. The vigorous use of the organs of mastication during the chewing of properly selected food will bring about an active circulation and stimulation of the parts involved, and, as a sequence, a rich flow of saliva required for the washing away of food debris and for the preliminary digestion of carbohydrate food is always insured.

Human saliva represents the mixed secretions from the three pairs of salivary glands and the minute mucous glands distributed over the oral cavity. Saliva may be defined as being a weak solution of alkalies, as present in the body juices, more or less saturated with carbon dioxid. It contains, furthermore, several organic substances, among which mucin and the several ferments which accelerate the changes of starches into maltose, *i. e.*, the hydrolysis of polysaccharids into soluble disaccharids. The ferments of human saliva are principally represented by the carbohydrate-splitting type, *i. e.*, amylase (ptyalin) and maltose, although oxydase and catalase are always present in more or less variable quantities. The physiologic function of mucin consists in mechanically assisting the food bolus in its easy passage into the stomach and to protect the oral tissues against irritating substances.

The biologic laws governing the secretion of saliva are directly responsible for its composition, its quantity, and its influence on the digestion and, incidentally, on dental caries. Only the most fundamental facts concerning these biologic aspects can be touched upon at this moment. The secretion of saliva depends upon nervous impulses. The quantity of saliva secreted, that is, the rapidity of its flow, depends upon the physical nature of the irritant (foodstuffs). Psychic stimulation is of less importance in this connection. Incidentally, the composition of saliva depends very largely upon the rapidity of flow, *i. e.,* its organic and inorganic contents are primarily the sequences as produced by the nature of the irritant. The irritant induces these changes, not merely in the oral mucous membranes, but also in those of the stomach. The latter seems to respond through the formation of hormones. Apparently, as has been shown experimentally by Pawlow and his pupils, saliva is a glandular secretion capable of adaption. However, the fundamental basis of the secretion of saliva rests with the process of mastication. The degree and the manner of mastication accelerates or diminishes very materially the nature of the irritant. The much discussed alkalinity of saliva depends directly on its ash contents; the more ash the higher the alkalinity. With an increase of the rapidity of flow an increase of alkalinity is always observed. Alkalinity of saliva as determined by titration is always a "one man's" finding and not to be relied upon To determine correctly the reaction of a fluid which, as saliva, hovers so closely near the neutral point, the electrometric measurement of the H-ion concentration is the only permissible scientific method. The writer has successfully employed for such work the gas-chain apparatus as modified by Michaelis. The reaction of normal saliva, *i. e.,*

saliva collected during periods of physiologic rest of the salivary glands is so very weakly alkaline that its influence as a so-called neutralizing medium of ''acidity'' of the mouth is practically nil. As the quality of saliva when collected during active digestion is always an expression of the nature of the last meal taken, saliva intended for analysis should be collected during resting hours of the digestive apparatus. The normal, healthy grown individual produces during waking hours approximately 1 c.c. of saliva per minute. During mastication, as stated above, depending upon the nature of the food stuff, this amount may be greatly increased.

Pickerill has made the assertion that the principal function of saliva consists in the hydrolysis of starches and thus prevents dental caries. The writer has not been able experimentally in the human mouth to show any relationship between the amylase (ptyalin) content of saliva and dental caries. Amylase may be readily paralyzed or accelerated by many chemic agents. Pure amylase is inactive as a ferment; certain inorganic ions, especially the chlorin ion (sodium chlorid), accelerates its activity ten times or more. Normal quantities, or even relatively large quantities of amylase may be, and occasionly are, present in the most rampant forms of caries. In certain animals, as for instance in the domestic dog, very little or no amylase is present in the saliva, although the dog is relatively immune to dental caries.

The much discussed bactericidal action of the saliva, which is claimed to be due to the presence of small and very variable quantities of potassium sulphocyanid, has been disproved by the classic researches of Miller, Bruylant, Gies and Kahn, and Kantorowicz. In a recent paper, McGehee attempts to revive the influence of sulphocyanids in its relation to the causation of dental caries, and, as a proof, he hypothetically quotes from Pauli's interesting little work: Physical chemistry in the service of medicine. McGehee has wholly missed his point. Dental caries does *not* depend on the living organism, and, as a consequence, the presence or absence of this chemical in metabolia processes as related to dental caries plays no part. In the normal mouth pathogenic micro-organisms are usually less virulent, than they are the subordinates of the saprophytic types. Fluegge has shown that the pathogenic bacteria will become extremely active if the individual is affiicted with a slight local disturbance—as a simple catarrh of the throat. Claermont has expressed similar views, and, after a careful study of the fluids of the mouth, he asserts that one is not justified in stating that saliva possesses any definite bactericidal action. It seems, however, that the parotid saliva of man and of some animals (especially the goat) exercises an inhibitory function on certain micro-organisms—the staphylococci and the streptococci.

Very recently an hypothesis relative to definite defensive or protective organisms possessed or produced by nature to combat the ravages of dental caries has been promulgated by McGehee. This conception is based on the ingenious experimental researches of Abderhalden, who holds the view that diseased processes are primarily regulated by defensive ferments. McGehee's conception is based on a misinterpretation of the Abderhalden theory. As we have stated above and wish to state again, dental caries is *not* a disease; it is a process of putrafaction, which may occur in a tooth, whether this tooth forms a part of the anatomy as a whole, or whether it is detached therefrom. The writer, two years ago, while working on this very question in Abderhalden's laboratory at the University of Berlin, convinced himself of the fact that defensive ferments, in the sense of Abderhalden's protective theory, play no part in the process of dental caries.

*The fermentative changes of the various types of saccharids into soluble sugars, the mechanical washing away*

*of accumulated food debris, and the ability of biologically inhibiting the virulence of pathogenic bacteria, are the important functions performed by a freely flowing saliva, and thereby maintain the physiologic equilibrium of the oral cavity.*

Immunity to dental decay, in the writer's opinion, depends—*caeteris paribus*—first, on a tooth free from imperfections of calcification, and, second, on a freely flowing saliva.

Immunity as referred to tooth structure is, in the strict sense of the word, a misnomer, as it is *not* bound up with vital phenomena. In a biologic sense, immunity indicates a state in which the "living" body resists disease. In a pulpless tooth, it goes without saying that we are dealing with dead structures as far as the enamel is concerned, and it is this latter tissue only that concerns us in the elucidation of the question: *Why* do teeth decay? In a tooth with a vital pulp, the writer holds the view that enamel is capable of carrying on metabolic processes to a limited degree. He is able to substantiate this claim by certain pharmacologic reactions, which, however, he can not discuss at this moment. The surface of so-called living enamel he regards practically as dead structure which offers no vital resistance to the physico-chemic process of decay. The omnipresent surface colloids and the colloidal fluids present in the enamel in teeth with living pulps modify the process of decay. All teeth which are imperfectly calcified on account of their lowered resistance will sooner or later decay until relative immunity is established in accordance with the imperative law of: Survival of the Fittest. If, however, the flow of saliva is impaired or completely checked, all teeth will be destroyed by caries unless some other means for the removal of food debris is established. The rapidity of the destructive processes is proportionally dependent upon the severity of the impairment. Normally, the flow of saliva is regulated by the intensity of the irritant as evinced during mastication. The stimulation by acids is of a temporary nature only, and of less importance. Therefore, vigorous mastication, or, as it is called by a recently popularized term: fletcherizing, of correctly selected food stuffs forms the basis for the natural prevention of dental decay.

To substantiate our contention relative to the position which saliva occupies in the prevention of caries, we may cite a few examples. In xerostomia, *i. e.*, inhibition of secretion of saliva, the teeth will begin to crumble away with the onset of the dry mouth. During other temporary pathologic disturbances of glandular activity, *i. e.*, continuous fevers (typhoid), menopause, diabetes, etc., clinically a marked increase of dental caries is always observed. The change of environments of food supply, *i. e.*, if the natural struggle for existence in gathering food is supplanted by artifically furnished food of a prepared type, a marked preponderance of dental caries, even in hitherto immune herbivorous animals, is always observed, as, for instance, in monkeys in captivity. The skulls of wild horses very rarely show carious defects; the domesticated horse is in frequent need of the veterinary dentist. Subjecting wild tribes of the human race to the influences of civilization, and, as a sequence, changed food supplies will always be followed by a most marked increase in dental caries. Immigrants from countries where hard-baked, black bread forms a large part of their staple diet, when coming to the United States are frequently subjected to intense ravages of dental decay. For instance, newly arrived Scandinavians, accustomed to chewing "knäckebröd," forget to masticate our soft wheat bread, and, as they are often neglectful of the blessings of the tooth brush, rampant decay is frequently manifest within a few months after landing. Dental caries is comparatively rarely observed in the teeth of habitual tobacco chewers. Miller and others have demonstrated that tobacco juice possesses no antiseptic action. Its prophylactic effect, as far as the teeth are concerned, rests with the pharmacologic action of tobacco, *i. e.,* its alkaloid, nicotin, is a powerful salivary stimulant.

In a recent communication (*Dental Cosmos,* 1913, p. 1081), Pickerill tentatively admits the importance of the *quantity* of salivary secretion. He states: I would even suggest that caries of the teeth may be regarded as a symptom of failure of the nervous mechanism controlling salivary secretions to functionate normally. In the writer's opinion, *the quantity of the secreted saliva is the sole factor which governs environmental phenomena concerning tooth decay.*

The quantity and, to a less extent, the quality of saliva, on account of our present methods of preparing and selecting foodstuffs and the consequent insufficient mastication, are frequently inadequate to bring about a proper physiologic cleansing of the oral cavity. To assist nature, suitable mechanical and chemical means may be employed to overcome this deficiency. The mechanical cleansing of the mouth and teeth by means of the brush, powder, paste, toothpick, floss silk, etc., constitutes the absolute fundamental principle of artificial oral hygiene. Food remnants and slimy adhesions between and upon the teeth, together with a large number of the adherent bacteria, are principally removed by mechanical cleansing. The mechanical cleansing of the oral cavity by these enumerated means may, however, be materially assisted by the judicious use of suitable mild astringent and indifferent antiseptic solutions. Powders, pastes and washes containing soluble drugs, or drugs in solution, are employed for the avowed purposes of assisting nature in accomplishing the desired means to an end, *i. e.,* they must favor the recovery of an inflamed mucous membrane, and they must mechanically remove accumulated food debris.

A good oral preparation should possess the following properties:

(1) It must be absolutely indifferent in regard to:

(a) the mucous membrane—*non-caustic.*

(b) the teeth—*non-decalcifying* (mechanical or chemical).

(c) the organism as a whole—*non-poisonous.*

(2) It must not interfere with the normal physiologic cleansing of that oral cavity, *i. e.:*

(a) it must not inhibit the secretion of saliva;

(b) it must not perceptibly alter the reaction of saliva;

(c) it must not destroy the ferments of saliva.

(3) It must possess sufficient cleansing action, combined with

(4) Good taste and odor.

These various enumerated properties are naturally rarely found in combination in a single oral preparation, and yet each one is of great importance.

Hygienic measures as applied to the oral cavity are practiced in proportion to the pleasant sensation which they call forth, hence, a mouth preparation which has a disgusting taste is ineffective because it will not be employed for any length of time by the laity. The great mass of the public will never be induced to practice oral hygiene that involves ill-tasting preparations. As stated above, mouth preparations must be absolutely free from danger as far as the mucous membrane, the teeth and the organism as a whole is concerned. Hence, Roese's dictum should be indelibly fixed in the mind of every dental and medical practitioner: The importance of oral antisepsis is not so great that we are justified in assuming the slightest risk. This statement can not be emphasized too strongly in view of the fact that numberless mouth washes and tooth preparations of questionable character are continuously forced on the market. Unless the correct composition of a ready-made mouth or tooth preparation is known, it should not be recommended.

The majority of the so-called dental preparations which are employed by the laity for daily use belong to a group of medicinal compounds generally known as proprietary preparations. As these compounds are not used for the avowed purpose of curing a specific disease, but rather as hygienic measures, no objection can be raised from an ethical point of view, provided that they are prepared from approved formulas and that they conform to the claims as outlined above.

A few of the more widely advertised preparations which are apparently universally recommended by the profession deserve special notice. Bad taste and general unfitness for the purpose in view are the lesser evils of most of these preparations; some are distinctly dangerous to the oral tissues when employed for daily use. The conception that mouth washes, tooth powders, and pastes which, in general, are non-poisonous and neutral in reaction, are indifferent to the oral tissues is erroneous. Many of these highly extolled compounds sail under dubious flags. For instance, an alkaline thymolated glycerin solution is claimed to possess extraordinary qualities as an oral antiseptic, while, in reality, it is about equally effective as a physiologic salt solution, but with a less pleasant taste. A fifty per cent. potassium chlorate tooth paste at one time furnished ''nature's antiseptic—free oxygen—which whitens the teeth,'' and, while at present it cures ''acid mouth,'' no trace of free oxygen was ever obtained from the use of this paste. The distinctive danger of potassium chlorate to the general health is, of course, not mentioned. Again, a mentholated salol solution is much lauded as "the most persistent oral antiseptic.'' This compound is rather prone to produce persistent eczematous eruptions about the corners of the mouth. Most of the widely advertised tooth powders, pastes and certain mouth washes contain too high percentages of soap. Soap, on account of its alkalinity, invariably kills the important salivary ferments. The list of ill-constructed mouth preparations may be extended *ad libitum*. In spite of the absurd claims made by the manufacturers, it seems incomprehensible that numerous practitioners recommend such compounds to their patients. The best service that a conscientious practitioner can render to his clientele is to absolutely prohibit the use of a mouth preparation of whose innocuousness he is not fully convinced.

The search for so-called tartar solvents as an addition to tooth preparations—substances which prevent or dissolve calcareous deposits about the teeth—has occupied the minds of the dental hygienists for some time past. The chemical nature of the oral calculus indicates that the logical solvent should be an acid or an acid salt. For plausible reasons, such substances can not be utilized in the mouth with impunity. It is known, however, that certain alkalies—the salines—prevent the ready formation of calculus, and they help to remove fresh deposits when brought in intimate contact therewith. Just how much of this destruction or removal should be attributed to the mechanical scrubbing of the brush, and how much to the solvent action of the ingredients of the tooth powder or paste, is not known at present. Nevertheless, the salts of certain mineral springs, especially those of Carlsbad, are used in concentrated form for such purposes, and apparently with some success. Artificial Carlsbad salts may be incorporated into a paste with calcium carbonate and other abrasives; its only drawback is its disagreeable salty taste. Tooth pastes containing about twenty-five per cent. Carlsbad salt may be obtained in the market.

Innumerable experiments have been made to determine the so-called antiseptic strength of oral preparations. As a standard, the Rideal-Walker phenol coefficient or some other laboratory standard is usually employed as a means of arriving at some tangible conclusions. If these experiments are carried out in test tubes with cultures of isolated organisms, comparative deductions drawn from such tests are wholly unwarranted, as they do not portray actual conditions existing in the oral cavity because the very premises upon which these experiments are based are erroneously chosen. On the other hand, if these preparations are tested directly in the mouths of normal individuals, it is invariably found that, in average, only fifty per cent. of the oral bacterial flora is inhibited. Authorities agree that it is impossible to render the oral cavity sterile, even for a short period only, with any of the so-far known antiseptic solutions (pastes, powders, etc., must enter into solution if any antiseptic effect is to be expected) in the strength in which these solutions can be employed with safety. The dilution of these preparations and the short time allowed for their action in the oral cavity as actually employed by the user, necessarily minimizes their antiseptic effect to such an extent as to practically render the solutions inert. Recently, Gies has advocated diluted vinegar and Pickerill a solution of acid potassium tartrate as being most efficacious mouth washes. Both recommendations are based on observations made in the laboratory; their correctness is not substantiated by clinical evidence. The recommendation of an acid mouth wash of the above type is based on wrong premises because, first, the laity will not be induced to employ an ill-tasting mouth wash for any length of time, and, second, the pharmacologic principle evolved in the selection of such solutions is erroneously applied. When an acid mouth wash in the form of vinegar or acid potassium tartrate is taken in the mouth, a temporary copious flow of alkaline saliva, rich in mucin, is produced. This alkaline saliva serves as a diluent and neutralizer of the acid and the colloidal mucin acts as a protector of the insulted mucous membrane and the teeth—nature's method of getting rid of the irritant. In accordance with Heidenhain's law, forcible stimulation of salivary glands is followed by impairment of their function. Incidentally, the acidity of these solutions kills the important salivary ferments. It has been repeatedly shown that a physiologic salt solution (approximately one dram of sodium chlorid to a pint of boiled water) heated to a body temperature, reduces the oral flora by fifty per cent. and, incidentally, it is absolutely safe. On the other hand, clinical evidence seems to point to the beneficial effects which are obtained by the use of such mild alkaline astringents as well diluted lime water. E. Kells, Jr., Kirk, and many other observers have repeatedly called attention to the remarkably good results obtained by its continuous use. Its therapeutic effect depends on its solvent power of the mucin deposits on and about the teeth, which mechanically retain food debris and bacteria and on the formation of insoluble soaps with fatty acids and lipoid substances. Incidentally, the freshly precipitated calcium carbonate may possibly exert some mechanical protective influences on the teeth themselves. When employed in proper dilutions, its mild astringent effect favors the recovery of inflamed mucous surfaces which, to a mild degree, are almost universally present in the mouths of most persons. A tablespoonful (one-half ounce) of lime water added to a tumblerful (eight ounces) of physiologic salt solution makes a most serviceable mixture which may be used as a mouth wash with impunity. Incidentally, this solution corresponds more closely to an artificial saliva—nature's protector of the teeth, than any other mouth wash found in the market.

The writer has made innumerable tests with the various dental preparations as found in the market and with experimental mixtures, by plating out specific quantities used within specific times in the oral cavity and counting the number of colonies before and after these tests. These experiments merely verify what has been stated above, namely:

1.—Sterilization of the oral cavity with any of the commercial dental preparations or any antiseptic, in the strength in which it can be employed with safety, can not be accomplished.

2.—The cleansing of the oral cavity with an antiseptic solution alone or combined with the mechanical effects of the tooth brush, powder, or paste, reduces the number of oral bacteria approximately about fifty per cent. The claims made for the antiseptic strength of certain commercial preparations are, by actual tests, wholly unwarranted.

3.—A physiologic salt solution of body temperature in conjunction with the tooth brush and precipitated calcium carbonate in the form of a powder or a paste (providing these preparations are in conformity with the claims as outlined above) is the safest and most efficient of all so far known, artificial, oral hygienic measures.—*Journal of Allied Societies.*

